# LRP6 High Bone Mass Characterized in Two Generations Harboring a Unique Mutation of Low‐Density Lipoprotein Receptor‐Related Protein 6

**DOI:** 10.1002/jbm4.10717

**Published:** 2023-03-02

**Authors:** Michael P Whyte, Steven Mumm, Jonathan C Baker, Fan Zhang, Homer Sedighi, Shenghui Duan, Tim Cundy

**Affiliations:** ^1^ Division of Bone and Mineral Diseases, Department of Internal Medicine Washington University School of Medicine at Barnes‐Jewish Hospital St. Louis MO USA; ^2^ Center for Metabolic Bone Disease and Molecular Research Shriners Hospitals for Children – St. Louis St. Louis MO USA; ^3^ Mallinckrodt Institute of Radiology, Musculoskeletal Section Washington University School of Medicine St. Louis MO USA; ^4^ Department of Plastic Surgery Washington University School of Medicine at St. Louis Children's Hospital St. Louis MO USA; ^5^ Faculty of Medical & Health Sciences University of Auckland Auckland New Zealand

**Keywords:** BONE DENSITY, BONE MODELING, DICKKOPF1, DXA, ENDOSTEUM, EXOSTOSIS, HYPEROSTOSIS, HYPODONTIA, LOW‐DENSITY LIPOPROTEIN RECEPTOR‐RELATED PROTEIN, *LRP4*, *LRP5*, *LRP6*, METABOLIC BONE DISEASE, OLIGODONTIA, OSTEOBLAST, OSTEOGENESIS, OSTEOPETROSIS, OSTEOSCLEROSIS, SCLEROSTIN, SKELETAL DYSPLASIA, *SOST*, TORUS PALATINUS, WNT, Β‐CATENIN

## Abstract

Osteoblast Wnt/*β*‐catenin signaling conditions skeletal development and health. Bone formation is stimulated when on the osteoblast surface a Wnt binds to low‐density lipoprotein receptor‐related protein 5 (LRP5) or 6 (LRP6), in turn coupled to a frizzled receptor. Sclerostin and dickkopf1 inhibit osteogenesis if either links selectively to the first β‐propeller of LRP5 or LRP6, thereby disassociating these cognate co‐receptors from the frizzled receptor. Sixteen heterozygous mutations identified since 2002 within *LRP5* and three heterozygous mutations identified since 2019 within *LRP6* prevent this binding of sclerostin or dickkopf1 and account for the exceptionally rare, but highly instructive, autosomal dominant disorders called LRP5 and LRP6 high bone mass (HBM). Herein, we characterize LRP6 HBM in the first large affected family. Their novel heterozygous *LRP6* missense mutation (c.719C>T, p.Thr240Ile) was present in two middle‐aged sisters and three of their sons. They considered themselves healthy. Their broad jaw and torus palatinus developed during childhood and, contrary to the two previous reports of LRP6 HBM, the appearance of their adult dentition was unremarkable. Skeletal modeling, defined radiographically, supported classification as an endosteal hyperostosis. Areal bone mineral density (g/cm^2^) of the lumbar spine and total hip featured accelerated increases reaching *Z*‐scores of ~ +8 and +6, respectively, although biochemical markers of bone formation were normal. © 2023 The Authors. *JBMR Plus* published by Wiley Periodicals LLC on behalf of American Society for Bone and Mineral Research.

## Introduction

Prominent among the rare Mendelian disorders manifesting generalized osteosclerosis and hyperostosis^(^ ^1^ ^)^ are the osteopetroses characterized over the past century^(^ ^2^ ^)^ and LRP5 and LRP6 high bone mass (HBM) recognized recently.^(^ ^3^ ^)^ The osteopetroses feature impaired osteoclast‐mediated skeletal resorption and are caused by loss‐of‐function mutations of genes necessary for osteoclast formation or action.^(^ ^2^ ^)^ Consequently, osteopetrotic bones fail to remodel, have poor quality, and often fracture.^(^ ^3^, ^4^ ^)^ In contrast, HBM features enhanced osteoblast‐mediated bone formation caused by selective heterozygous mutations of *LRP5* or *LRP6* that alter the function of their encoded proteins, low‐density lipoprotein receptor‐related protein 5 (LRP5) and 6 (LRP6), respectively.^(^ ^3^, ^4^ ^)^ On the osteoblast surface, when a Wnt couples to either of these cognate co‐receptors^(^ ^5^ ^)^ complexed with a frizzled receptor, canonical Wnt/β‐catenin signaling increases and generates good‐quality bone.^(^ ^6^, ^7^ ^)^ Physiologic inhibition of LRP5 or LRP6 occurs if either sclerostin or dickkopf1 binds to its first β‐propeller, thereby disassociating it from the frizzled receptor.^(^ ^8^, ^9^, ^10^, ^11^ ^)^ This inhibition is lost if the β‐propeller is defective. Despite the contrasting pathogenesis underlying the osteopetroses versus LRP5 HBM or LRP6 HBM, encasement of teeth as well as compression of the brain and one or more cranial nerves are potential complications from the excessive bone.^(^ ^2^, ^4^ ^)^

LRP6 HBM was first identified in 2019, and only two small families^(^ ^12^ ^)^ and one sporadic occurrence^(^ ^13^ ^)^ are reported. Herein, we characterize LRP6 HBM affecting five people in two generations of a Māori family in New Zealand who carry a unique fourth *LRP6* defect.

## Subjects and Methods

**Fig. 1:**
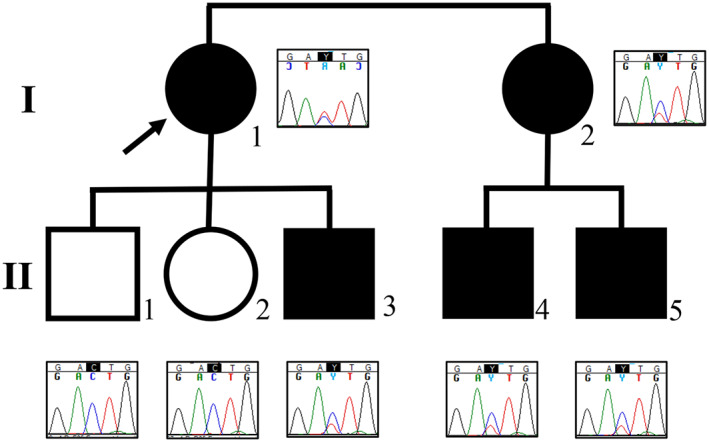
Family pedigree. Proposita 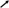; 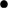 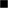 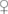, 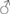 with *LRP6* high bone mass (HBM). The electropherograms distinguish the *LRP6* missense mutation. Co‐segregation analysis supported the *LRP6* defect as pathogenic (*p* < 0.0008).

The proposita was incidentally discovered at age 43 years to have generalized osteosclerosis and hyperostosis consistent with a heritable bone disorder. Her sister and their five children then underwent evaluation (see Results), revealing five affected individuals, ie, the two sisters and three of their sons (Fig. 1).

The electropherograms distinguish the *LRP6* missense mutation. Co‐segregation analysis supported the *LRP6* defect as pathogenic (*p* < 0.0008).

### Proposita

The proposita (I‐1: Fig. 1) presented with a 10‐month history of neuralgic pain in her neck and right arm. Radiological studies (see below) revealed a dense skeleton. Magnetic resonance imaging (MRI) demonstrated spinal stenosis from disk prolapse at cervical vertebrae 5/6 and 6/7. Computed tomography (CT) showed generalized hyperostosis of the skull and facial bones. No intracranial pathology or cranial nerve compression was apparent. Surgical decompression of the prolapsed disks improved her symptoms, but disk or bone histology was not undertaken. She had fractured her left distal radius, including its growth plate, with significant trauma at age 9 years. After several weeks of casting, the wrist grew asymmetrically. Its alignment was improved by surgery at age 19 years. Trauma also explained her tooth loss. For many years, there had been intermittent headaches. Two years earlier, she had complained of achy knees and hands and a possible diagnosis of rheumatoid arthritis had been considered. Antinuclear antibodies were present in low titer; other serologies were negative. She and her elder affected nephew (II‐4; Fig. 1) said they cannot float, and instead “sink.” She recalled no previous mention of dense bones.

**Fig. 2:**
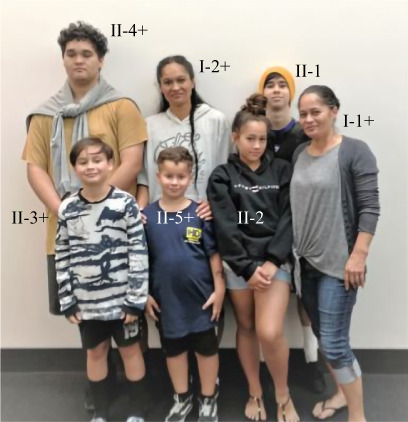
Proposita and other family members. Mandible widening becomes apparent during childhood for those with LRP6 high bone mass (HBM) (designated +).

**Fig. 3:**
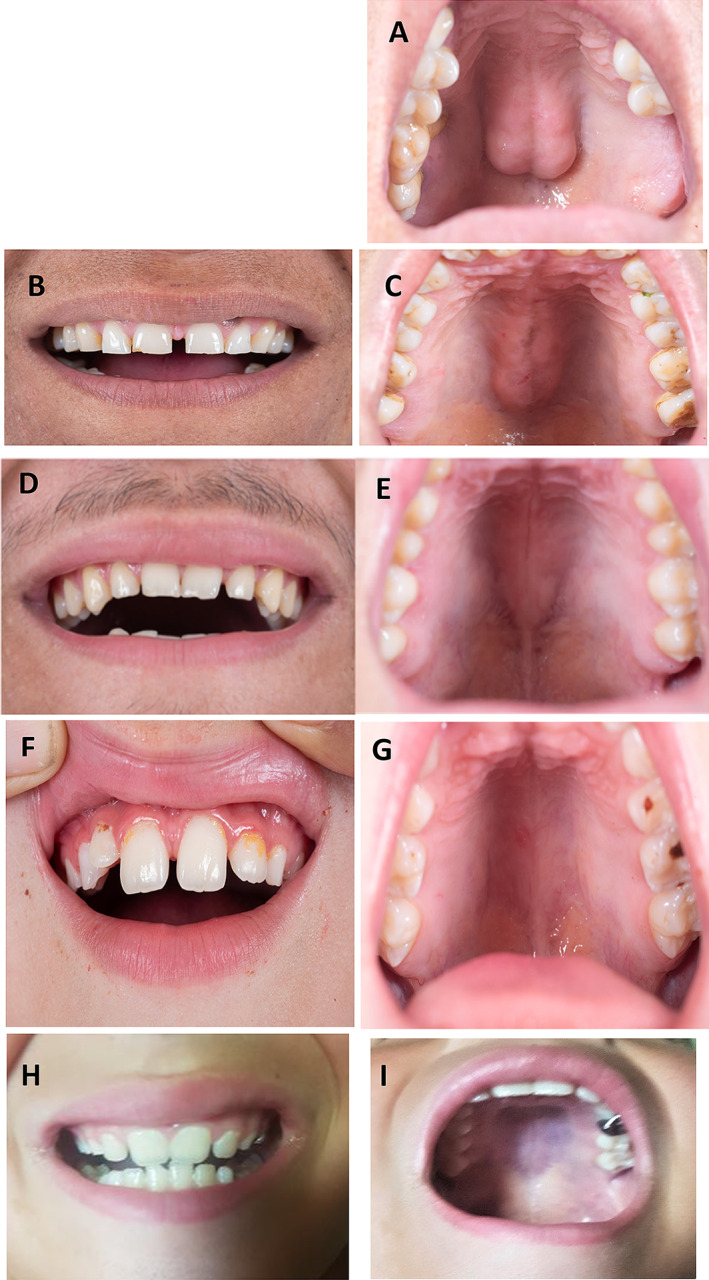
Teeth and hard palate of family members with LRP6 high bone mass (HBM). (*A*) I‐1 (proposita), age 44 years, shows torus palatinus and tooth loss from past trauma. There are no retained primary teeth. The dental morphology and anatomy of her permanent teeth are normal. The enamel appears solid with bright white coloration and no obvious caries. (*B*, *C*) I‐2, proposita's 41‐year‐old sister, has a torus. All maxillary permanent teeth are present. There are no retained primary teeth or apparent caries. Diastema is observed between teeth #8 and #9. Enamel is solid bright white. (*D*, *E*) II‐4, age 15 years, has a small torus. All maxillary permanent teeth are present, with no retained primary teeth or dental caries. There is generalized maxillary anterior teeth spacing but no posterior interdental spacing. The maxillary left central incision has a minor enamel fracture/chip, possibly from an injury or bruxism. (*F*, *G*) II‐3, age 10 years, has no torus. Primary and secondary dentition are present. No carious lesion is apparent. There is normal wearing of the primary teeth. The permanent maxillary right and left central incisions look elongated. No inflammation or bruxism is apparent. (*H*, *I*) II‐5, age 7 years, has mixed dentition with no torus. The primary maxillary left first molar was treated/restored using a stainless steel crown, indicating a previous carious lesion. The LRP6 HBM in this family is not associated with congenital hypodontia or exostoses surrounding teeth. Only commonplace dental changes are present. Torus palatinus develops during childhood (compare *G* and *I* with *A* and *C*).

Her height was 160 cm (63 inches), weight 65 kg (143 lbs), and body mass index (BMI) 25.4 k/m^2^. She had a broad jaw (Fig. 2) and large palatal torus (Fig. 3) but no other exostoses. Her left forearm was mildly deformed. Cranial nerve examination was normal, but audiometry was not performed.

**Fig. 4:**
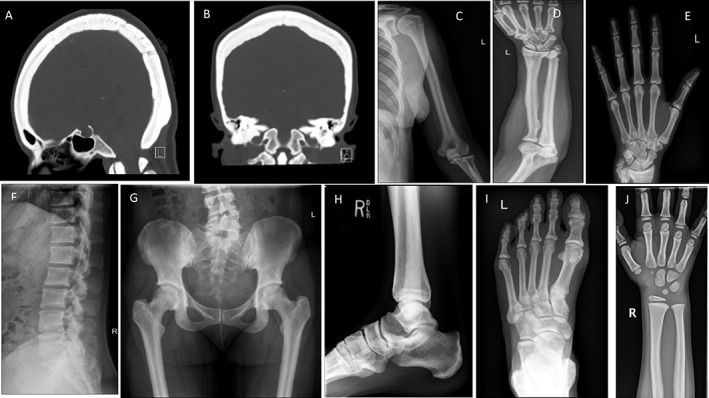
Radiological findings. (*A*, *B*) Sagittal and coronal CT image of the head using bone windowing shows severe calvarial thickening with hyperostosis of the inner and outer tables and effacement by osteosclerosis of the normal diploic space trabecular pattern. The upper cervical spine also manifests dense osteosclerosis in the C1 ring and C2 odontoid process. The frontal sinus is hypoplastic. (*C*–*E*) Anteroposterior (AP) radiographs of the left humerus, forearm, and hand show osteosclerosis of the humerus, radius, ulna, and long bones of the hand. They have thickened cortices with narrowed medullary canals. The middle third of the radius has mild but non‐specific periosteal thickening. Elsewhere, long bone size, shape, and general formation are unremarkable. Healed fracture deformities are present in the distal radius and ulna. The joint spaces are normal, except for post‐traumatic osteoarthritis at the distal radioulnar joint. (*F*) Lateral radiograph of the lumbar spine shows mild osteosclerosis of the vertebral bodies and posterior elements with mild, multisegmental, degenerative disk disease and facet osteoarthritis. There is no endplate thickening to suggest a “rugger jersey” spine or compression deformity. (*G*) AP pelvic radiograph shows diffuse hyperostosis and osteosclerosis with narrowed medullary spaces in the proximal femora. Corticomedullary differentiation is maintained, and there are no growth arrest lines. Decreased height of the right portion of the L_5_ vertebral body may be developmental or from chronic degenerative remodeling. (*H*, *I*) Lateral radiograph of the right foot and dorsoplantar radiograph of the left foot, respectively, show generalized osteosclerosis and cortical thickening. A small, bony excrescence arises from the right navicular bone. (*J*) II‐3, when age 6 years, shows mild osteosclerosis on AP view of his right wrist.

Because her family seemed well and all would undergo (see below) dual‐energy X‐ray absorptiometry (DXA) screening (GE Lunar DPX, Madison, WI, USA), only she was evaluated with a radiographic skeletal survey. It revealed diffuse osteosclerosis and endosteal hyperostosis (Fig. 4).

DXA showed areal (g/cm^2^) bone mineral density (aBMD) in her L_1_ to L_4_ spine 2.107 g/cm^2^ (*Z*‐score + 7.7); left total hip 1.849 g/cm^2^ (*Z*‐score + 6.9); and left femoral neck 1.725 g/cm^2^ (*Z*‐score + 5.1), respectively; ie, 79%, 90%, and 84% above mean aBMD for age‐matched women.

### Family study

After genetic analyses that revealed the proposita's LRP6 HBM (see below), six additional family members (Fig. 1) were screened for this diagnosis during a single outpatient visit, which included routine clinical, biochemical, and DXA assessments, and leukocyte DNA acquisition for *LRP6* mutation analysis (see below). A radiograph of II‐3, from age 6 years, showed mild osteosclerosis (Fig. 4). Reportedly, the proposita's mother had torus palatinus.

### Mutation analyses

Initially, the proposita's leukocyte DNA was evaluated for diagnosis in a commercial laboratory (Blueprint Genetics, Seattle, WA, USA) using version 3 of their “Osteopetrosis and Dense Bone Dysplasia Panel Plus.” Sequence and copy number variation analysis did not examine *LRP6*, but instead *LRP5* as well as *AMER1*, *ANKH*, *CA2*, *CLCN7*, *COL1A1*, *CTSK*, *DLX3*, *FAM20C*, *CJA1*, *LEMD3*, *OSTM1*, *PTDDS1*, *PTH1R*, *SLC29A3*, *SLCO2A1*, *SNX10*, *SOST*, *TCIRG1*, *TGFB1*, *TNFRSF11A*, *TNFRSF11B*, *TNFSF11*, and *TYROBP*. No mutation was identified. Therefore, in our laboratory, following informed written consent approved by the Human Research Protection Office, Washington University School of Medicine, St. Louis, MO, USA, we amplified by PCR and Sanger‐sequenced exclusively *LRP6* exons 2–4 encoding the LRP6 first β‐propeller. We designed the primers^(^ ^12^ ^)^ (available upon request). Subsequently, DNA from each family member was Sanger‐sequenced, blinded to their clinical information, selectively for the proposita's *LRP6* missense mutation (see Results). Genes involved further in the WNT/β‐catenin pathway were not assessed.

### Statistical analyses

Height *Z*‐scores were calculated using the SAS program of the US Centers for Disease Control and Prevention (CDC) growth chart website (https://www.cdc.gov/nccdphp/dnpao/growthcharts/resources/sas.htm) for sex‐matched individuals 0 to 20 years of age. For the proposita and her sister, reference information was based on the data from the 20‐year‐old control women.

Regression analysis was chosen to explore if LRP6 HBM spine, total hip, or femoral neck aBMD (g/cm^2^) was associated with age. Subsequently, we also considered: (i) aBMD data added from our two previous reports of LRP6 HBM^(^ ^12^, ^13^ ^)^ and then separately (ii) our experience^(^ ^12^ ^)^ and other relevant publications^(^ ^14^, ^15^, ^16^, ^17^, ^18^, ^19^, ^20^, ^21^, ^22^ ^)^ concerning aBMD in LRP5 HBM. Statistical analyses and graphics utilized SAS software 9.4 (SAS Institute Inc., Cary, NC, USA). Two‐sided *p* < 0.05 was deemed statistically significant.

## Results

### *LRP6* mutation analysis

**Fig. 5:**
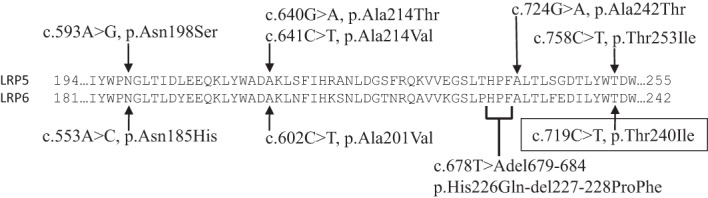
Amino acid sequence and mutations of *LRP5* and *LRP6*. Amino acid alignment of LRP5 and LRP6 first β‐propeller regions where homologous high bone mass (HBM) mutations overlap. Five of the known 16 *LRP5* mutations are shown with the homologous four *LRP6* mutations. *The family's *LRP6* mutation is boxed.

Sanger sequencing selectively of the proposita's *LRP6* exons 2–4 showed in exon 4 a unique heterozygous missense variant (c.719C>T, p.Thr240Ile). This variant is not reported in the gnomAD browser as of October 2022.^(^ ^23^ ^)^ Likewise, none of the three previously published *LRP6* HBM mutations are reported in gnomAD. Using PolyPhen2 (Polymorphism Phenotyping v2), this new variant is predicted to be “probably damaging” with a score of 0.968 (sensitivity: 0.77; specificity: 0.95).^(^ ^24^ ^)^ Using the sorting intolerant from tolerant (SIFT) algorithm, this variant is predicted to “affect protein function” with a score of 0.00.^(^ ^25^ ^)^ Further, the family's variant (c.719C>T, p.Thr240lle) is precisely homologous (same nucleotide and amino acid change) to a defect associated with LRP5 HBM reported in 2003 (Fig. 5).^(^ ^26^ ^)^ Subsequently, the proposita's sister, two nephews, and younger son were found to harbor the same variant, whereas her elder son and daughter did not. The *LRP6* variant co‐segregated (*p* = 0.0008) with their elevated bone density identified by DXA (Fig. 1). These findings support *LRP6* c.719C>T, p.Thr240Ile as pathogenic according to the American College of Medical Genetics (ACMG) guidelines.^(^ ^27^ ^)^

### Family findings

The family's key demographic, clinical, DXA, and biochemical findings, discussed below, are summarized in Table 1.

**Table 1:** LRP6, Demographic, Clinical, DXA, and Biochemical Findings of Family Members

Subject^a^	*LRP6* mutation	Age/sex	Height (cm, percentile, *Z*‐score)	Fractures	Hearing/eyes	Torus	Other	Spine *Z*‐score	Total hip *Z*‐score	Femur neck *Z*‐score	L_1‐_L_4_ aBMD (g/cm^2^)	Calcium (corrected)^b^ mmol/L	Phosphate^c^ mmol/L	ALP^d^ U/L	P1NP^e^ μg/L
I‐1	+	44 ♀	160 (40) (−0.25)	[L] ulna age 9 years – surgery age 19 years	Normal	Yes	Cervical stenosis; headaches	+7.7	+6.9	+5.5	2.107	2.37	1.29	82	39
II‐1	−	15 ♂	163 (31) (−0.50)	No	Good	No		+0.3	−0.1	−0.7	1.010	2.38	1.44	263	648
II‐2	−	13 ♀	151 (17) (−0.95)	[R] elbow age 8 years	Reading glasses	No	Dyslexia	+1.8	+1.4	+1.9	1.112	2.38	1.82	260	611
II‐3	+	10 ♂	131 (14) (−1.1)	[L] wrist age 6 years	Grommets age 6 years	No	Bifid uvula	+2.5	+4.7	+ 4.8	0.989	2.35	1.45	295	737
I‐2	+	41 ♀	176 (98) (+2.05)	None	Good	Yes	Rheumatic fever as a child	+7.9	+5.4	+4.2	2.126	2.33	1.33	60	38
II‐4	+	16 ♂	193 (>97) (+1.88)	None	Keratoconus age 15 years	Early		+5.4	+4.8	+5.8	1.866	2.40	1.33	101	326
II‐5	+	7 ♂	140 (>97) (+1.88)	None	Normal	No		+5.8	+3.8	+4.2	1.143	2.35	1.52	208	709

^a^ Yellow shaded area reports the two family members without the *LRP6* mutation.

^b^ Serum calcium. Normal range: 2.15–2.55 mmol/L.

^c^ Serum phosphate. Normal ranges: 1.06–1.70 mmol/L, ages 5–15 years; 0.8–1.4 mmol/L, ages >15 years.

^d^ ALP (alkaline phosphatase). Normal ranges: 80–500 U/L, ages 5–10 years; 60–450 U/L, ages 11–16 years; 40–130 U/L, adult.

^e^ P1NP (procollagen‐1 N‐propeptide). Normal ranges: 250–800 μg/L, ages 5–10 years; 380–1050 μg/L, ages 11–16 years; 20–80 μg/L, adult. Measured by electrochemiluminescence (E170, Roche Diagnostics, Mannheim, Germany).

#### Clinical phenotype

The family considered themselves healthy. One unaffected (II‐2) and two affected (I‐1, II‐3) family members had suffered a single upper‐limb fracture during childhood. None considered swimming difficult. Routine clinical examination as well as evaluation of photographs of the teeth and hard palate revealed torus palatinus in the two affected women (I‐1, I‐2) and in an early form in the affected 16‐year‐old son (II‐4) but not in the two youngest affected sons (II‐3, II‐5) (Fig. 3) or in their unaffected siblings (II‐1, II‐2). There was no congenital hypodontia, exostoses surrounding teeth, or other noteworthy^(^ ^12^, ^13^ ^)^ dental findings. Three of the five affected individuals (I‐2, II‐4, II‐5), but neither of those unaffected (II‐1, II‐2), were tall (ie, >97th centile), consistent with our experience concerning both LRP5 and LRP6 HBM (Table 1).^(^ ^12^ ^)^

#### DXA findings

Elevated lumbar spine, total hip, and femoral neck aBMD *Z*‐scores were found exclusively in all five family members harboring the *LRP6* mutation. The tall stature of the three affected sons (I‐2, II‐4, II‐5) perhaps artifactually increased, but slightly, their aBMD (g/cm^2^) values. The aBMD *Z*‐scores of the proposita and her sister averaged nearly +8 in the lumbar spine and +6 in the total hip (Table 1).

**Fig. 6:**
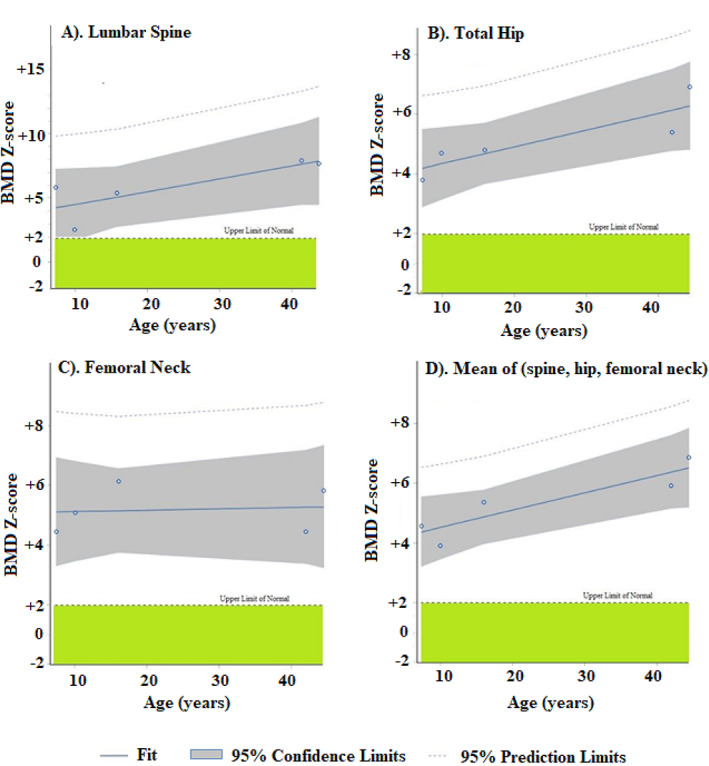
Fit plots of areal bone mineral density (aBMD) *Z*‐scores versus subject ages. All five family members with LRP6 high bone mass (HBM) have elevated aBMD *Z*‐scores in their (*A*) lumbar spine, (*B*) total hip, and (*C*) femoral neck (green bar indicates ±2 SD control mean). These *Z*‐scores in the lumbar spine and total hip, but not femoral neck, appear to increase with age. (*D*) Using the mean of each subject's three site‐specific *Z*‐scores supports this correlation of increasing aBMD with age (*p* = 0.0366).

Linear regression analysis indicated the aBMD *Z*‐scores individually for the lumbar spine and total hip, but not in the femoral neck, increased approximately +2 SD from childhood to middle age. This finding was then supported by regression analysis using the mean values of the combined *Z*‐scores of these three skeletal sites versus age (Fig. 6). Our prior observations^(^ ^12^ ^)^ for LRP6 HBM suggested a significant positive association of increasing aBMD *Z*‐scores in the lumbar spine but not total hip and for LRP5 HBM no such association.^(^ ^12^ ^)^ Now, pooling all of our LRP6 HBM data,^(^ ^12^, ^13^ ^)^ and separately published data^(^ ^12^, ^14^, ^15^, ^16^, ^17^, ^18^, ^19^, ^20^, ^21^, ^22^ ^)^ concerning LRP5 HBM, supported such associations with increasing age (Supplemental Appendix S1).

#### Biochemical findings

Mineral homeostasis of all family members was normal, including normal serum calcium and phosphorus levels. Parathyroid hormone, measured only in the proposita, was normal. In those individuals with LRP6 HBM, serum alkaline phosphatase and procollagen type 1 N‐terminal peptide levels were age‐appropriate despite their sourcing from elevated skeletal mass (Table 1).

## Discussion

Below, we briefly review the discovery, nosology, genetic basis, and pathogenesis of LRP5 HBM and LRP6 HBM and then consider their phenotypic similarities and perhaps differences.

### Discovery, nosology, and etiology

What we now call HBM has been described in the medical literature for at least one‐half century, but its expanding taxonomy has impeded recognition and understanding. In 1966, HM Worth and DJ Wollin^(^ ^28^ ^)^ suggested *hyperostosis corticalis generalisata congenita* for this autosomal dominant disorder, aiming to distinguish it from autosomal recessive van Buchem disease (*hyperostosis corticalis generalisata familiaris*).^(^ ^29^ ^)^ Subsequently, the term “Worth‐type endosteal hyperostosis,” correctly emphasizing the radiographic hallmark of endocortical bone thickening, became widely adopted.^(^ ^30^, ^31^ ^)^ However, starting in 1986, some reports used the designation “autosomal dominant osteopetrosis, type 1” (ADO1),^(^ ^32^ ^)^ although osteoclast failure is not the principal pathogenetic feature,^(^ ^6^ ^)^ yet this term persists (OMIM # 607634).^(^ ^30^ ^)^ We have endorsed^(^ ^12^, ^31^ ^)^ “HBM,” coined by Johnson and colleagues^(^ ^33^ ^)^ in 1997 when they linked the LRP5 HBM phenotype to chromosome 11, but recognize that LRP6 HBM must now be distinguished from LRP5 HBM. In 2022, Bergen and colleagues^(^ ^34^ ^)^ proposed an ontology based upon the biological process and/or pathway for monogenic disorders that feature elevated bone mass. Following uniform Gene Ontology terminology, LRP6 HBM would fit within the subgroup 24 that includes LRP5 HBM, sclerosteosis (types 1 and 2), van Buchem disease, craniodiaphyseal dysplasia, and several other heritable disorders that involve dysregulation of WNT signaling.^(^ ^34^ ^)^

**Fig. 7:**
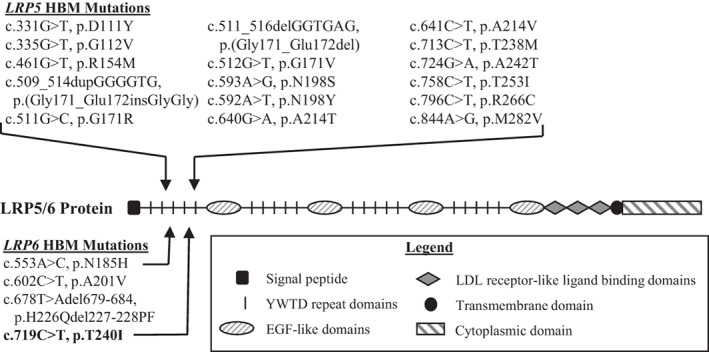
*LRP5* and *LRP6* mutations causing high bone mass (HBM). LRP5/6 protein showing HBM mutations. *LRP5* mutations are shown above the protein and *LRP6* mutations are shown below. The legend shows LRP5/6 domains. Each series of YWTD repeat domains comprises a β‐propeller.

The genetic basis of LRP5 HBM was discovered by two groups.^(^ ^14^, ^17^ ^)^ In two American kindreds in 2002, the identical heterozygous missense mutation was identified in *LRP5* (c512G>T, p.Gly171Val). Initially, LRP5 HBM was considered “non‐syndromic”^(^ ^14^ ^)^ by lacking signs, symptoms, or complications, although buoyancy was poor during swimming. In the second kindred,^(^ ^17^ ^)^ the “syndromic” broad jaw and torus palatinus of affected individuals was emphasized. However, in 2004, we reported a young American woman carrying the identical *LRP5* mutation^(^ ^15^ ^)^ but suffering cranial nerve palsies, Chiari I malformation, diffuse skeletal pain, and dental exostoses, thereby revealing that LRP5 HBM can be a dento‐osseous disease. Several publications have further characterized LRP5 HBM^(^ ^4^, ^12^, ^14^, ^15^, ^16^, ^17^, ^18^, ^19^, ^20^, ^21^, ^22^, ^35^, ^36^ ^)^ and disclosed 16 causal heterozygous mutations that disrupt the first β‐propeller of LRP5 (Fig. 7).^(^ ^14^, ^15^, ^16^, ^17^, ^18^, ^19^, ^20^, ^22^, ^26^, ^35^, ^36^, ^37^, ^38^, ^39^, ^40^, ^41^ ^)^

We discovered LRP6 HBM in 2019^(^ ^12^ ^)^ when evaluating two small American families with a disorder closely resembling LRP5 HBM but lacking an *LRP5* mutation and perhaps adding congenital hypodontia to the phenotype. Then, in 2020, we reported LRP6 HBM with dental anomalies, including hypodontia, in an Argentine college student.^(^ ^13^ ^)^ To date, three heterozygous mutations in *LRP6*, all disrupting the protein's first β‐propeller, have been associated with HBM (Fig. 7). Below, we discuss the fourth such defect.

### LRP5 and LRP6 HBM pathogenesis

HBM, van Buchem disease (OMIM # 239100),^(^ ^30^ ^)^ and sclerosteosis types 1 and 2 (OMIM #269500, #614305),^(^ ^30^ ^)^ all considered “endosteal hyperostoses,” have in common a pathogenesis that involves deficient inhibition of bone formation by sclerostin.^(^ ^3^, ^4^ ^)^

LRP5 and LRP6 proteins are 70% identical and 83% homologous (excluding amino acids 1–30 of LRP5 and 1–18 of LRP6).^(^ ^12^ ^)^ LRP5 and LRP6 are single‐pass transmembrane proteins with multiple domains. They bind Wnt ligands as well as sclerostin and dickkopf1.^(^ ^36^, ^37^, ^38^ ^)^ At their N‐terminus, the first 24 amino acids of LRP5 and the first 19 of LRP6 are the signal peptide that enables them to cross the plasma membrane.^(^ ^26^, ^39^ ^)^ The extracellular portions include four tandem YWTD‐type β‐propeller domains, which precede an epidermal growth factor (EGF)‐like domain, which precedes three LDLR type A domains. The YWTD positions and sequences are highly conserved, but less so the LDLR repeats essential for ligand binding. LRP5 and LRP6 couple related, but not necessarily highly similar, ligands,^(^ ^42^, ^43^, ^44^, ^45^ ^)^ including many Wnts (eg, Wnt1, Wnt2, Wnt2b, Wnt6, Wnt8a, Wnt9a, Wnt9b, and Wnt10b) that interact with the first β‐propeller domain.

Three of the now four LRP6 HBM mutations affect amino acids that are also implicated in LRP5 HBM (Fig. 5). Two *LRP6* missense mutations (c.602C>T, p.Ala201Val, and the one carried by the current family c.719C>T, p.Thr240Ile) are precisely homologous to mutations causing LRP5 HBM.^(^ ^26^, ^39^ ^)^ The third *LRP6* missense mutation (c.553A>C, p.Asn185His) affects the homologous amino acid position, with a different amino acid change.^(^ ^39^ ^)^ The fourth *LRP6* defect (c.678T>Adel679‐684, p.His226G1n‐del227‐228ProPhe) is adjacent to a homologous *LRP5* mutation.^(^ ^13^ ^)^

Biallelic mutations affecting LRP5 elsewhere than its first β‐propeller cause the autosomal recessive disorder called osteoporosis‐pseudoglioma syndrome (OMIM # 259770)^(^ ^30^ ^)^ featuring severe osteoporosis and blindness.^(^ ^46^ ^)^ LRP6 is present in many tissues. *Lrp6*, but not *Lrp5*, null mice die at birth,^(^ ^47^ ^)^ suggesting *Lrp6* has the greater prenatal importance. Biallelic mutations of *LRP6* in humans are presumably embryonically lethal, therefore yet to be reported.

Heterozygous *LRP5* mutations, elsewhere than the first β‐propeller, may cause low bone mass, sometimes ocular complications,^(^ ^48^ ^)^ but also mesiodens, tooth agenesis, root malformation, and taurodontism.^(^ ^49^ ^)^ Such heterozygous variants in *LRP6*, too, have been associated with early‐onset osteoporosis, although less often than similar *LRP5* variants.^(^ ^50^ ^)^ *LRP6* single‐nucleotide polymorphisms have been linked with high or low aBMD.^(^ ^51^, ^52^ ^)^ Furthermore, heterozygous mutations of *LRP6* elsewhere than its first β‐propeller have been associated with: (i) tooth agenesis with “oligodentia” (OMIM # 616724)^(^ ^30^, ^53^, ^54^, ^55^ ^)^ and rarely (ii) neural tube defects.^(^ ^56^ ^)^ Most of the 16 *LRP6* mutations associated with oligodontia alter the second or third β‐propellers, whereas most associated with neural tube defects cluster in the second β‐propeller or the intracellular domain.^(^ ^56^ ^)^ *LRP6* mutations elsewhere than affecting the first β‐propeller have also been associated with early‐onset atherosclerosis.^(^ ^57^, ^58^, ^59^ ^)^ Of interest, Pickering and colleagues^(^ ^60^ ^)^ in 2021 reported a young woman with skeletal changes resembling Camurati‐Engelmann disease (OMIM #131300),^(^ ^30^ ^)^ who carried a novel *LRP6* defect that would compromise the second β‐propeller, and Puente and colleagues^(^ ^61^ ^)^ in 2022 reported familial low bone mineral density associated with a mutation in the second propeller region. These *LRP6*‐associated disorders did not seem present in the family with LRP6 HBM we studied herein.

### Clinical comparison of LRP5 and LRP6 HBM

Reflecting the cognate co‐receptor function of their encoded proteins, LRP5 and LRP6 HBM phenotypes are remarkably similar. In the family herein, the five individuals with LRP6 HBM considered themselves healthy, although some reported low‐level bone pain or arthralgias, yet were “syndromic” with acquired broad jaws and torus palatinus. In LRP5 HBM, it seems the age when torus palatinus appears is uncertain. The LRP5 HBM literature concerns 20‐ to 77‐year‐old people, with torus reported in 19 of 24 (79%); the five without this exostosis were 23 to 76 years of age. Beals suggested^(^ ^62^ ^)^ in LRP5 HBM^(^ ^26^ ^)^ that with age the torus “increases slowly in size,” but later commented that “the age when torus palatinus appears has not been documented.”^(^ ^63^ ^)^ Our findings suggest that the torus palatinus of LRP6 HBM begins in adolescence. It was uncertain if the proposita's cervical disk prolapse and arthralgias were related to her LRP6 HBM. In each of our two prior reports of LRP6 HBM,^(^ ^12^, ^13^ ^)^ one episode of transient facial nerve palsy had affected a child but was explained by trauma^(^ ^12^ ^)^ or infection.^(^ ^13^ ^)^ Persisting cranial nerve compression has not complicated LRP6 HBM, although MRI of the Argentine student showed subtle stenosis of the optic canals and narrow internal auditory canals.^(^ ^13^ ^)^ Above average height seems characteristic and similar in both LRP5 HBM and LRP6 HBM.^(^ ^12^ ^)^ Thus far, our impression is that exostoses that obscure especially the posterior teeth, absent on routine physical examination of the family herein, are less severe in LRP6 HBM compared with LRP5 HBM.^(^ ^15^, ^16^ ^)^ Hypodontia (particularly congenital absence of the maxillary lateral incisors) has occurred in LRP6 HBM^(^ ^12^, ^13^ ^)^ but not in LRP5 HBM. However, the family herein reveals that oligodontia, too, is not invariable in LRP6 HBM, and perhaps previous associations were coincidental because absence of adult maxillary lateral incisors is a prevalent autosomal dominant form of oligodontia.^(^ ^30^ ^)^

Mineral homeostasis seems intact in LRP5 HBM and LRP6 HBM. Bone turnover markers are in the high‐normal range, but perhaps this reflects their sourcing from elevated bone mass.

In 2019, we reported^(^ ^12^ ^)^ the principal radiographic features of both LRP5 HBM and LRP6 HBM were: (i) generalized osteosclerosis, (ii) uniformly thickened diploic space in the skull (sometimes so dense there is no trabecular pattern), (iii) poor development (aeration) of the frontal and maxillary sinuses, (iv) radiodense orbital roofs and facial bones, (v) mandible with obtuse angle and rounded body, (vi) teeth obscured by exostoses, (vii) submental process protrusion, (viii) mild shaping defects of long bones but with endosteal hyperostosis, and (ix) diffusely sclerotic vertebral bodies without superior or inferior end plate accentuation (ie, no “rugger‐jersey spine” appearance). Our proposita manifested many such features. In either LRP5 HBM or LRP6 HBM, joint spaces are generally preserved despite osteosclerosis that in autosomal dominant “adult” (“benign”) osteopetrosis, caused by heterozygous *CLCN7* mutations, is implicated in osteoarthritis.^(^ ^64^ ^)^

The aBMD *Z*‐scores in the lumbar spine range from +3.1 to +12.2 (LRP5 HBM) and similarly from +2.5 to +10.3 (LRP6 HBM), and at femoral sites from +2.4 to +12.2 (LRP5 HBM) and similarly from +3.8 to +10.1 (LRP6 HBM). Analysis of all 13 individuals with LRP6 HBM indicates spinal, but not femoral, aBMD *Z*‐scores increase with age (Supplemental Appendix S1). Further study will disclose if perhaps elevated aBMD (g/cm^2^) in both LRP6 HBM and LRP5 HBM plateau at young adult life.

Currently, LRP5 HBM and LRP6 HBM seem remarkably similar, emphasizing why mutation analysis is key for differential diagnosis. Now, *LRP6* analysis should be considered for unexplained generalized elevation of bone density. Our experience indicates good health in LRP6 HBM, at least until middle age, but study of affected elderly will be especially significant because LRP action is multifaceted and important. In LRP6 HBM, the increasing aBMD *Z*‐score in the lumbar spine during growth may be concerning or may plateau and be protective against age‐related bone loss. The significance of understanding LRP6 HBM and LRP5 HBM has been increased by recent availability of monoclonal antibodies against sclerostin to treat low bone density, especially if given to children.

## Disclosure

The authors declare no conflicts of interest.

## Author Contributions

All authors approved the submitted manuscript. MPW arranged for mutation analysis of LRP6, performed by SD and assessed by SM, and then developed this report.  JCB described the radiological findings.  HS interpreted the photographs of the family’s dentition.  Biostatistician FZ helped identify the association of bone density with patient age.  TC selected, evaluated, and summarized the testing that identified and characterized the family’s LRP6 HBM, and then reviewed background literature and data.

### Peer Review

The peer review history for this article is available at https://publons.com/publon/10.1002/jbm4.10717.

## Supporting information


**Appendix S1.** Supplementary Files
